# Spouses' existential loneliness when caring for a frail partner late in life - a hermeneutical approach

**DOI:** 10.1080/17482631.2020.1734166

**Published:** 2020-03-01

**Authors:** Helena Larsson, Margareta Rämgård, Christine Kumlien, Kerstin Blomqvist

**Affiliations:** a Faculty of Health and Society, Department of Care Science, Malmö University, Malmö, Sweden; b Faculty of Health Sciences, Kristianstad University, Kristianstad, Sweden

**Keywords:** Existential loneliness, spouses, frail partner, hermeneutics, multistage focus group interview, poems

## Abstract

**Purpose**: Spouses are in a vulnerable situation when caring for a frail partner late in life. Exploring their existential loneliness can be a way to understand more about their existential needs.

**Method**: A hermeneutic approach was used. Multistage focus group interviews were conducted with two groups consisting of five spouses, respectively, who met three times each. To work with the text, an approach was adapted where quotations are converted into poems in a linguistic manner.

**Results**: Existential loneliness can be understood as the following: 1) being in a transition from *us* to merely *me*, 2) being forced to make decisions and feeling excluded, 3) navigating in an unfamiliar situation and questioning oneself, and 4) longing for togetherness but lacking the energy to encounter other people. The main interpretation is that existential loneliness emerges when one is in moments of inner struggle, when one is forced to make impossible choices, when one is approaching and is in limit situations, and when one is experiencing the endless loss of the other.

**Conclusion**: For health care professionals to achieve a holistic picture, person-centeredness can be a way to make the spouses’ existential needs visible and to provide support based on their needs.

## Introduction

Existential loneliness seems to occur especially in vulnerable situations (Sjöberg, Beck, Rasmussen, & Edberg, 2017). One such situation can be caring for a frail partner in the last phase of life. Studies describe how existential loneliness appears when close relationships with other people change (Larsson, Rämgård, & Bolmsjö, 2017; Sjöberg et al., 2017). Another aspect is that spouses experience feelings such as guilt, loneliness (Hogsnes, Melin-Johansson, Norbergh, & Danielsson, 2014), isolation (Greenwood & Smith, 2016; Neri et al., 2012) and aloneness (Førsund, Skovdahl, Kiik, & Ytrehus, 2014) when their partner becomes increasingly impaired. To enhance the well-being of spouses who care for a frail partner, it is important to understand their existential concerns, where one such concern can be experiences of existential loneliness.

According to Yalom (1980), life involves circumstances when we face existential loneliness, such as our need for freedom and belongingness, our search for meaning, and the inevitable death. Existential loneliness has been compiled in literature reviews as a complex phenomenon (Ettema, Derksen, & van Leeuwen, 2010; Mayers & Svartberg, 2001) intertwined with feelings of emptiness and nothingness (Ettema et al., 2010). A conceptual analysis (Bolmsjö, Tengland, & Rämgård, 2018) reveals how existential loneliness emerges as an immediate and instantaneous perception that we, as human beings, are fundamentally alone, separated from other people. This perception appears especially in times when we become aware of our own mortality or are in a crisis, especially when we are unfamiliar with or not met by others in the situation. Accordingly, negative feelings such as sadness, hopelessness, anxiety and meaninglessness are experienced (Bolmsjö et al., 2018). Existential loneliness has also been empirically studied in different health care contexts, for example, in the care of people with aphasia (Nyström, 2006), among women living with HIV (Mayers & Svartberg, 2001), in relation to end-of-life care (Sand & Strang, 2006), among frail older people (Sjöberg et al., 2017) and their significant others (Larsson et al., 2017), and health care professionals (Sundström, Edberg, Rämgård, & Blomqvist, 2018). One conclusion that can be drawn from these studies is that existential loneliness is a phenomenon that emerges in vulnerable situations and involves a range of feelings and expressions. Listening to the voices of spouses who care for a frail partner late in life may be one way of learning more about existential loneliness.

Research reveals that one vulnerable situation for a couple late in life is when one of them has to be relocated from their own home to a care home. One study from the UK focused on informal caregivers’ experiences, and it describes how the relocation impacted their emotional well-being, where feelings of guilt, helplessness and failure to be able to continue care were prominent (Milligan, 2005). A Swedish study illustrates how spouses experienced guilt and loneliness both before and after relocation (Hogsnes et al., 2014). According to a review from the UK, informal carers over the age of 75 experience a tension between both rewarding feelings and difficult feelings: rewarding when feeling supported by the municipality and having a social network, difficult when feeling isolated and having their own health problems (Greenwood & Smith, 2016). Another review compiles spouses’ experiences of living with a partner diagnosed with dementia. It shows that the everyday life together changes on all levels, where the overall theme is the irreversible loss of their relationship with their partner (Pozzebon, Douglas, & Ames, 2016). The loss of a shared couplehood made spouses feel alone and disconnected, not only from their partner but also from society in general (Førsund et al., 2014). A quantitative study presents how 115 out of 176 older informal carers evaluated caregiving as stressful and isolating (Neri et al., 2012). When one partner in a togetherness becomes frail, this put demands on the other partner. The situation is, for example, defined as “Living in the presence of death”, where existential concerns—such as existential distress, death anxiety and uncertainty—are revealed (Melin-Johansson, Henoch, Strang, & Browall, 2012). An empirical study shows how spouses see themselves as followers to the end of life and that they adapt their everyday life to the needs of the dying person (Andersson, Ekwall, Hallberg, & Edberg, 2010). Understanding existential loneliness from different perspectives, such as that of the spouses’, can be a way to discern more about the spouses’ situation and existential needs.

The current study is part of a larger research project about existential loneliness (Edberg & Bolmsjö, 2019), where one recent study focused on older persons’ existential loneliness as interpreted by their significant others (Larsson et al., 2017). During the interviews with those who were spouses, we gained a sense of how they had their own experiences of existential loneliness linked to living close to a frail partner. This led us to the current study. Therefore, the aim of this study was to explore spouses’ existential loneliness when caring for a frail partner late in life.

## Method

The LONE study, RR2-10.2196/1307 (Edberg & Bolmsjö, 2019) is in the development phase of designing a complex intervention (MRC, Medical Research Council, 2008) where existential loneliness is explored through interviews with frail older people (Sjöberg et al., 2017), their significant others (Larsson et al., 2017), and health care professionals (Sundström et al., 2018). The concept “frail” was defined, according to the LONE study, as older (>75) persons, late in life and dependent on long-term care or services related to health problems.

### Design

A hermeneutic approach (Gadamer, 1960/1990) was chosen to explore spouses’ experiences of existential loneliness when they care for, or had cared for, a frail partner. To explore the experiences in-depth, multistage focus group interviews were conducted (Hummelvoll, 2008). The research strategy has a dialogical nature characterized by the same group exploring a topic in depth through several meetings. The researcher decides the topic and functions as a moderator throughout the whole process (Hummelvoll, 2008). In the present study, two focus groups met three times each, which made it possible to follow up narrations about existential loneliness through the multiple meetings. Meeting spouses in a group, instead of individually, enables the participants to reflect upon each other’s experiences (Krueger & Casey, 2015). Hermeneutics is described as an art of interpretation, and working with the text is essential (Dahlberg, Dahlberg, & Nyström, 2008; Gadamer, 1960/1990). In order to work with the text, an approach used by, among others, Schuster (2006) and Edvardsson, Sandman, and Rasmussen (2006), was adapted where quotations are converted to poems in a linguistic manner (Gee, 1985, 1991). The purpose of using this particular poetically technique was to facilitate what is difficult to describe, thereby giving the reader a better sense of existential loneliness.

### Setting

Nearly 1 million persons in Sweden, approximately 10% of the population, are 75 years or older (SCB, Statistics Sweden, 2018). The care of older people is primarily a municipal responsibility and is mainly financed by taxes. The formal care is provided by 290 municipalities, and relatives who care for a family member at home have a legal right to support, according to Swedish law (Socialtjänstlagen, 2001:453, 5:10 [Law about Social services]). The support includes, for example, health care and/or service in the home, daily activities arranged by the municipalities, or part-time care at a nursing home. The informal care involves about 1.3 million of the Swedish population (SCB, Statistics Sweden, 2018). In the current study, two municipalities were involved.

### Sample and procedure

The sample consisted of ten spouses: five men and five women. Inclusion criteria were as follows: have experiences of living together with and caring for a frail partner; consider themselves and their partner as a couple for a long time; and see themselves as the principal responsible for their partner. The first group included spouses whose partner was still alive (n = 5), while the second (n = 5) included those who had lost their partner within the five last years. Two of the participants, belonging to the first group, became a widow/widower during the data collection period of five weeks. All ten spouses had experiences of living together with and caring for a frail partner, seven had experiences of losing their partner, and five had experiences of how life became after their partner had died. For a description of the sample, see Table I.

**Table I.** Description of the sample

Informants	n = 10
Men/women	5/5
Age, median (range)	79.5 year (67–89 years)
Lived together, median (range)	51.5 years (46–65 years)
Widow/widower, median (range)	3/2, 2 years (1–4 years)
Partner’s main concern: Dementia Cancer Other	6 3 1
Partner’s care contact: Living/lived at home Municipal home care Municipal home service Specialized palliative home care Residential care	5 3 3 3 5
Lived in the same municipality, median (range)	47 years (5–86 years)
If something happens, do you have anyone to contact?	All answered yes
Do you have anyone who relieves/d you? Yes No Has/had no need	5 1 4

A coordinator in the municipality was contacted and informed about the study through a physical meeting with the first author (HL). The coordinator acted as a link between the researchers and potential spouses, for example by recruiting spouses to participate in the study and asking for permission to communicate their name and telephone number to HL. In addition, the coordinator provided potentially interested spouses with a letter of information about the study. The letter contained information about the aim of the study, a description of existential loneliness as a deeper feeling of loneliness, and a guarantee of confidentiality. For informants to feel comfortable talking about and sharing their experiences, each group was to consist of 4–6 participants (Krueger & Casey, 2015). In total, 15 names and numbers were communicated to HL, 10 of whom agreed to participate. Reasons for not taking part in the study were an unwillingness to talk about the topic, the informants’ own health problems, or not answering the phone, despite repeated calls.

### Data collection

Data were gathered by using two focus groups, who met three times each, with each session lasting approximately two hours. The sessions took place between August and October 2018. There was a two-week interval between session one and two, and a three-week interval between session two and three, with each session recorded and transcribed verbatim. All of the sessions were conducted in a room provided by the municipality, with the group collectively deciding the day and time of the sessions. Both focus groups were kept intact for the duration of all three sessions, and all of the participants, except for one exception in each group, took part in all sessions.

**Figure 1.**
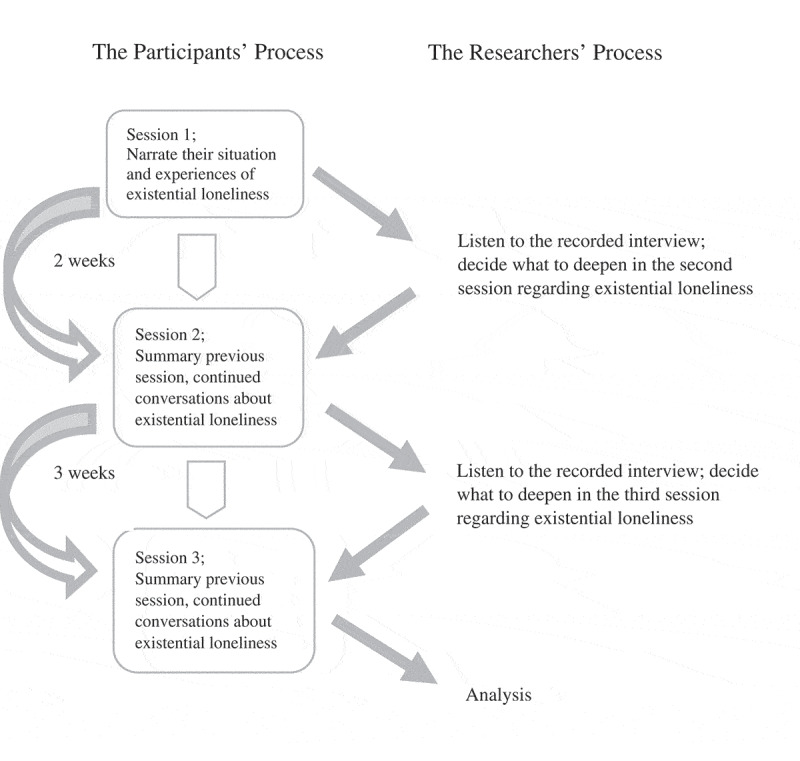
Description of the procedure during data collection

The focus groups were led by HL as moderator and the last author (KB) as an observer. All sessions commence with brief information about the aim of the study. According to Hummelvoll (2008), the first session should serve to create a good atmosphere and let participants become familiar with each other, which was done through allowing all participants to narrate their situation as a spouse. Thereafter, the opening questions (Hummelvoll, 2008) concerned loneliness, in general, and existential loneliness, in particular: How do you experience loneliness in your situation right now? In relation to that, what could you say about existential loneliness? Members in the group who had lost their partner were asked to narrate how they experienced existential loneliness when they had cared for their partner in relation to its present state. Members of both groups were encouraged to describe their experiences in narratives and to reflect upon their own stories and those of others. After each session, the moderator and the observer listened to the recording to review the content (Hummelvoll, 2008) and decided what to deepen regarding existential loneliness for the next session. For a description of the procedure during data collection, see Figure 1.

### Analysis

When all six sessions were completed, a structure for a hermeneutical analysis by Dahlberg et al. (2008) was used. All data were read as a whole text by HL and KB to ascertain a preliminary understanding. Tentative themes emerged and covered the following: changes in life, being left behind, questioning oneself and others, and the absence of togetherness. The tentative themes were presented for all authors, and the second and third author read the data to verify the content. After establishing themes, meaning units (not too short, rather a whole narrative) were extracted (Dahlberg et al., 2008). Dialogue as a means of working with a text (Gadamer, 1960/1990) is exemplified by Schuster (2006) and Edvardsson et al. (2006), whose approach we adopted. It concerns working with a text in a linguistic manner through converting quotations into poems (Gee, 1985, 1991). The poem is constructed by choosing a central quote, i.e. a text that clearly illustrates the essence of the theme; reading it slowly; dividing the quote into stanzas, usually four lines; notice pauses, false starts, hesitations; and considering the meaning of each word in order to catch the essence of the text (Gee, 1985, 1991; Schuster, 2006). The process facilitated the interpretation because a poem is read slowly; for example, as one’s breathing becomes calmer when reading poetry (Rämgård & Nieminen Kristofersson, 2010; Schuster, 2006), the poem is read with another sense. Only the quotations presented in this paper were converted into poems. When poems were established for each of the four themes, the writing commenced to enable the reader to understand how the interpretation had progressed (Schuster, 2006). Finally, a main interpretation was conducted to create an understanding of the whole (Dahlberg et al., 2008; Gadamer, 1960/1990).

During analysis and in the interviews, it was important to be aware of the pre-understanding. A hermeneutic horizon of understanding emphasizes that pre-understanding should be considered an unavoidable fact and be used as an asset (Gadamer, 1960/1990). This was done by continually discussing and comparing the interpretations with our pre-understanding. Because the four authors have different professional backgrounds—including caring science, geriatric nursing, surgical care and human geography—it was possible to look at data with different perspectives.

### Ethical considerations

The study was approved by the Ethical Review Board, Lund, Sweden (Reg.no. 2018/422). The first session, in both groups, commenced with information about ethical issues, such as voluntary participation and confidentiality. All participants signed an informed consent. Thereafter, all three sessions started with a reminder of voluntary participation.

## Findings

Spouses’ existential loneliness when caring for a frail partner can be understood according to four themes: 1) being in a transition from *us* to merely *me*, 2) being forced to make decisions and feeling excluded, 3) navigating in an unfamiliar situation and questioning oneself and, 4) longing for togetherness but lacking the energy to encounter other people.

### *Being in a transition from* us *to merely* me

Being in a life-changing event that is not self-chosen and not possible to influence causes the emergence of existential loneliness. The text describes a longing for life together as it once was and, simultaneously, an awareness that the changes that occur are irreversible and permanent, and that life will never ever be the same again. In the poem below, we follow a husband’s experience when he begins to feel that something is not right with his wife.

I did not recognize her

passive

sat on the couch

[M: Do you miss her?]

Yes!

how she was before

oh, yes

I can handle it yet

but

slowly but surely it will become worse

before we have complemented each other well

but now it is not so good anymore

unfortunately

The first part of the poem covers how it was *then*, when the man realized that his wife changed to the unrecognizable. In the last part, he is in the present, and the change in the relationship emerges. Previously, they “complemented” each other, but not any longer. The text describes uncertainty for the future with the words “slowly but surely worse” and sadness in the word “unfortunately”.

Another example of the transition is the use of language. The text continues to talk about *us* and *me*. In the poem below, we follow the conversation between two women talking about how they now are supposed to speak: whether they should say *I* or *us*?

It does not happen by itself

you have to imagine it

I would also like to say *we*

and the *thinking*

To think

I

instead of *we*

yes

and always this with *we* inside the mind

The text describes how they have always imagined themselves as one, as a *we*, but now they need to start to think of themselves as merely *me*. To imagine merely me as I, instead of we, does not come spontaneously; rather it needs a directed, conscious thought. The words “does not happen by itself” talk about how strange it is to imagine me, as only I, instead of we. Similarly, the words “inside the mind” relate that a transition takes time. Living as we is a deeply rooted feeling, and it takes time to change the image from *we* to only *me*. The text continues with narratives about feeling like a half person when “we” no longer exists. In the poem below, we follow a woman in her narrative a time after her husband’s death, a time when she can look back at “then”.

Emptiness

but somehow

a relief

but for the most part

a great emptiness

After fifty years together

I felt like

a half person

I did

Since there is a description of relief, we can imagine that there has been a time of anguish, otherwise the word relief would never have had to be used; thus the relief was preceded by anguish. Likewise, we can understand from the words “a half person” that the feeling of being a whole person has existed previously. To go from feeling like a whole person to feeling like a half person means we can understand that losing one’s life companion is a crucial event in a person’s life. It involves to live in a transition from *us* to merely *me*.

### Being forced to make decisions and feeling excluded

To surrender a life-companion into the hands of others is considered as an impossible choice. However, it is a decision the spouses feel obligated to make, and it is one that causes the emergence of existential loneliness. A decision must be made even though no alternative is satisfying. The choice is not only devastating but also crucial for the rest of their lives. In the poem below, we follow a woman’s illustration of how it was the very first time she entrusted her husband to part-time care at a nursing home.

When I left him the first time

for the very first time

worried about how it would be like

I know how he is at home

And I was instructed not to visit him during that following week

and neither did I

I picked him up later

And then I thought

maybe I might try to have him at home anyway

I do not want to expose myself to this

The text deals with the spouses surrendering their partner into care for the first time as a decisive stage. On the one hand, spouses do not want to make decisions, but, on the other hand, doubts are expressed—as with the word “instructed”—about the decisions of others. Doubts about their own decisions are also expressed with “I might try”. Thus, the wife considered the possibility of caring for her husband at home a little longer. That the situation is perceived as painful is made clear at the end of her depiction with the words “I do not want to expose myself to this”.

The text concerns not only experiences of how it is to surrender a partner to care but also how difficult it is to be the one who left is behind, thereby becoming excluded. To be forsaken and excluded cause existential loneliness to surface. In the poem below, we follow a man’s portrayal of how it was when his wife excluded him.

I had looked after her

during the nights

all of the time

and helped

And then Friday night

she asked for herself

a nurse to arrange something

it went very quickly

It was the most difficult day for me

she moved away from *our* home

through her own free will

it was heart-breaking

She wanted to move away from the home we had built up together for sixty years

she thought she was getting better help

at another place

It was difficult

I knew

she would not come back

The text contains the words “through her own free will” and is understood as the man feeling forsaken by his wife. After having taken care of her day and night, she chooses to leave *him*. His narration conveys that no matter how much he did for her he was not enough because she thought that she was getting better help from someone else. Expressions such as “it was heart-breaking” and “difficult” encompass that to be forsaken and excluded is a painful situation. At the end of the poem, the awareness of the definitive separation is expressed by the words “I knew, she would not come back”.

### Navigating in an unfamiliar situation and questioning oneself

To be in an unfamiliar situation without knowing how to navigate causes existential loneliness to surface. In the poem below, we follow a woman describing her unfamiliar situation with the metaphor of being in a bubble, as in a vacuum.

Like to be in a bubble

I know that someone called

I answered and they said

How are you?

Well, I said

I do not know

I think I am in some kind of bubble

I do not know

trapped

in a vacuum

Then, you are alone

you do not really know what to do

in this bubble

The bubble was horrible

after all, it has limits

it has been like a vacuum

Words such as “it has limits” express feeling disconnected from a context. The bubble is referred to as “horrible”, so the feeling is something unpleasant, something to be avoided. Being in the bubble is a situation of ambivalence, hesitation and, simultaneously, a paralysis because it is portrayed with the words “you do not really know what to do”. The bubble is also referred to as “then, you are alone”, so the bubble has a delimited time frame that stays in one’s mind. In the poem below, the depiction continues, partly with the same woman’s reflection, partly at the end with another woman’s confirmation that it is difficult to understand another person until you, yourself, have been there.

It is strange, you know

it is also strange, based on what I have worked with

I have worked with people all of my life

You never become immune

when it happens to you, it is something other

than what you have seen others experience

and you have believed that you have understood

The text deals with the fact that though having previously thought or having said to others that they understand, they now realize how they do not understand, not until they themselves experience something similar. The word “strange” shows that in the woman’s self-reflection, she is surprised: she had not expected the situation to feel like this. The text reveals that it is painful to question oneself in a self-reflection. At the end of the poem, the text relates “you have believed that you have understood”, where the word “believed” shows that the woman now realizes that she has not understood what people previously experienced. In the text, the words “you never become immune” show how feelings and situations like this are difficult to defend oneself from. The endeavour in life can be to protect oneself, to become immune, but it is impossible to become immune to difficult situations such as being trapped in a bubble, delimited in a vacuum. This is one kind of situation that can be understood as navigating in an unfamiliar situation and as questioning oneself.

### Longing for togetherness but lacking the energy to encounter other people

Having previously, for several years, belonged to a given context with another person who is now transforming to the unrecognizable or is lost through death causes the emergence of existential loneliness. The text describes how difficult it can be to find a new context and, at the same time, how difficult it can be to remain in the previous contexts when no longer a couple. In the poem below, we follow a wife in her description of how it was when her husband moved to a nursing home and they celebrated their first Christmas together in a new context.

We celebrated Christmas up there with them

a horrible experience

to sit with half-dead people and celebrate

awful

nobody talked

only me

The text relates that *we* celebrated Christmas with *them*. The absence of belongingness and togetherness is total and is expressed in words such as “half-dead people” and “nobody talked”. The words “only me” can be understood as if the woman felt lonely even though the room was full of people. Expressions such as “horrible” and “awful” indicate that this is a context she does not want to be in. She lives with her husband but cannot feel togetherness with him or his context.

The text continues with depictions of a longing for togetherness with other people, especially when the partner no longer lives. In the poem below, we follow a woman in her longing to be close to other people and to share her thoughts with others and, simultaneously, how difficult it can be to have the energy to do so.

When I am at home

I just wander

cry

I feel *this* [pointing with both hands towards the chest]

very concrete

So, in order to disperse *this* [pointing with both hands towards the chest]

I have to get out

but then again, that togetherness

that I might experience for a while

it lasts only a little while

then I have to go home behind my closed door anyway

But then

you may not have the energy to take part in that gathering

I slipped out through the back door

I felt that I could not

could not be a part of that gathering that should be a given for me

The text conveys a longing for togetherness, but, at the same time, a struggle to find the energy to encounter other people. This is related through the notion of trying to meet other people while slipping out through the back door and not wanting to be alone at home and wandering and being reminded of the emptiness but, simultaneously, not being able to stay away. The word “this” together with both hands pointing towards the chest becomes an expression of the emptiness that appears when the context and the togetherness with a life-companion is broken forever.

### Main interpretation

The main interpretation is that existential loneliness emerges when one is in moments of inner struggle, when one is forced to make impossible choices, when one is approaching and is in limit situations, and when one is experiencing the endless loss of the other. The themes can be comprehended as if the spouses are in a process that leads to the inevitable final destination in their relationship with their partner. With descriptions such as “I felt like a half person”, the poems can be explained and understood as if the experience is like that of being torn apart. The process can be understood in line with Jaspers’s (1994) description of limit situations, where an unavoidable limit has been reached which the spouses can neither escape from nor defend themselves from. A previous study has shown that existential loneliness can be understood as a feeling of being totally separated from other people (Bolmsjö et al., 2018). The interpretation of limit situations can be understood through poems as being trapped in a bubble that has limits. Another explanation of “the bubble” is found in Yalom’s (1980) metaphor of existential isolation, illustrated as a valley. To face dying and death inevitably leads to the valley. Some of the poems also allow us to understand that limit situations have a definite time frame. The poems describe a clear direction, with words like “this” or “then”. The direction, therefore, indicates that the spouses know exactly where the feeling is inside, but that they find it difficult to put into words. Instead, the hands are used to show the direction of the feeling, for example, pointing with both hands towards the chest. The direction is also about time: the time of being in the bubble, in the ambivalence—the limit can clearly be described to a delimited time. This has been addressed in the study by Bolmsjö et al. (2018), where existential loneliness is described as an immediate and instantaneous perception: right in that moment, probably impossible to escape from for the spouses. The poems can also be understood as if the spouses feel alone in their struggle, and we can assume that they do not feel encountered at a deeper level, which is described as a situation that causes existential loneliness to emerge (Bolmsjö et al., 2018).

In the themes, situations of choice appear as central. In the poems, we can understand that the spouses pose constant questions, such as “If I do like this—will it be better for him then?” and, on the other hand, “If I do like that—would it be better?” The spouses experience that they have no choice; rather, they feel compelled to make decisions that are not satisfying, such as surrendering their partner to a nursing home or accepting the change that takes place with their partner. The poems can be understood as if the spouses are in an inner struggle with themselves. They want to do good; but whatever they choose to do, their choice will not be satisfying for either themselves or their partner. To be in this battle is to experience existential loneliness. Tillich (1952/2000) asserts that everyone is in a constant tension between oneself and the world. The choices we make are not satisfying, but not to choose is, as Sartre (1947/2007) claims, also a choice. It is unpleasant to be forced into a choice that causes anxiety. Tillich (1952/2000) maintains that constant, inevitable choices cause anxiety, and an expression of existential loneliness is anxiety (Bolmsjö et al., 2018).

A contemporary philosophy is person-centeredness, which has its starting point in each person’s unique desires and a strengthening of trust in their own choices (McCormack & McCance, 2017). To support the spouses in their choice is important. All of the spouses’ partners are provided with continuous care. Both involving and listening to the spouses through the process of care is crucial (Søvde, Hovland, Ullebust, & Råholm, 2018). Studies highlight the significance of supporting, listening to and catering for the needs of close relatives who, in various ways, care for a family member (*cf*. Totman, Pistrang, Smith, Hennessey, & Martin, 2015). Accordingly, preventive work should also centre on those who are in the patient’s absolute vicinity, that is, to include them in a person-centred care. However, person-centred care can be distinguished as including only the person in need of care. The spouses in the present study have lived in a togetherness for 50 years, they are *one*, as a unit, rather than two separate individuals. Jaspers (1971/1995) relates the importance of looking at the whole situation, because if we only look at certain parts, the picture becomes fragmented and impossible to perceive correctly; thus it becomes skewed and misleading. Actively targeting the idea towards a holistic picture, that is person-centeredness (McCormack & McCance, 2017), can be a way to make the spouses’ own needs visible.

### Methodological considerations

To strengthen the dependability (Shenton, 2004), variation in the sample was required. Consequently, we aimed to reach not only those spouses who had care responsibilities at that time but also those whose partner had died within the last five years. The sample involves spouses who have lived with their partner for a long time. As the median is 51.5 years, it can be seen as a measure for sharing experiences from a relational perspective. The method, multistage focus group interviews (Hummelvoll, 2008), was chosen because we wanted to reach a deeper reflective dialogue. We also strove towards an atmosphere built on trust as we wanted the participants to talk about what can be construed as a difficult topic. Therefore, it was vital to meet the participants several times over a prolonged period. During the data collection, we were one junior researcher and one senior researcher, both of whom were familiar with conversations about existential issues. In order for the reader to verify (Shenton, 2004) the analysis, each step is described in the method, in a figure and in poems. The results are presented in such a manner that it is possible for the reader to verify how the interpretation progressed. To safeguard confirmability, the awareness of our preunderstanding was important (Shenton, 2004), where one measure is our different professional backgrounds. Regarding the transferability (Shenton, 2004), the informants were identified from two municipalities and their partner was given care from different care contexts; this may strengthen the transferability.

### Conclusion and clinical implications

The main value of this study is a deepened understanding of existential loneliness when caring for a frail partner late in life. The main interpretation is that existential loneliness emerges when one is in moments of inner struggle, when one is forced to make impossible choices, when one is approaching and is in limit situations, and when one is experiencing the endless loss of the other. The spouses are in a vulnerable and demanding situation. For health care professionals to accommodate and support spouses in their demanding situation, knowledge and understanding of existential concerns are important. To achieve a holistic picture that includes acknowledging people’s physical, social, emotional and existential needs, person-centeredness can be a way to make the spouses’ existential needs visible by listening and being present. In addition, a topic for further research is to develop existential support specifically targeted to spouses who care for a frail partner late in life.
